# A Novel Nanocomposite of Zn(II)-Protoporphyrin-Chitosan-Multi Walled Carbon Nanotubes and the Application to Caffeic Acid Sensing

**DOI:** 10.3390/nano12193412

**Published:** 2022-09-29

**Authors:** Bingkai Han, Xin Wen, Jinneng Wang, Yingrui Sun

**Affiliations:** 1 Tianjin Key Laboratory of Exercise Physiology and Sports Medicine, College of Exercise Health, Tianjin University of Sport, No. 16 Donghai Road, West Tuanbo New Town, Jinghai District, Tianjin 301617, China; 2 The Key Laboratory of Bioactive Materials, Ministry of Education, College of Life Science, Nankai University, Weijin Road No. 94, Tianjin 300071, China

**Keywords:** metalloporphyrin, caffeic-acid biosensor, multi-walled carbon nanotubes, chitosan

## Abstract

Caffeic acid is an antioxidant that has been widely been related to the health benefits of people in recent years. In this paper, the amino side chains of chitosan (CS) were modified with protoporphyrin IX by amide cross-linking, and then Zn ions were chelated. The properties of metalloporphyrin-preparing functionalized multi-walled carbon nanotubes (MWCNTs) and Zn ions chelated by protoporphyrin IX composites were used as sensitive-selective electrochemical biosensors for the determination of caffeic acid. The morphology and structure of nanocomposite Zn–PPIX–CS–MWCNTs were observed by X-ray spectroscopy mapping (EDX mapping), transmission electron microscopy (TEM), and Fourier-transform infrared spectroscopy (FTIR). The electrochemical behaviors of Zn–PPIX–CS–MWCNT-modified glassy carbon (GC) electrodes were evaluated by cyclic voltammetry (CV) and differential pulse voltammetry (DPV). The results show that the modified electrode had good electrocatalytic activity towards caffeic acid with a wide linear range of 0.0008–1.6 mM, an excellent sensitivity of 886.90 µAmM^−1^cm^−1^, and a detection limit of 0.022 µM. In addition, the caffeic acid sensor had excellent reproducibility, stability, and selectivity to various interfering substances. Therefore, the modified electrode prepared by this experiment can also be applied to electrochemical sensors of other substances.

## 1. Introduction

Caffeic acid is a polyphenolic acid widely found in food plants [1]. It is a polyphenolic acid widely distributed in vegetables, fruit, coffee, tea, wine, and other substances. It has many biological functions and is easily absorbed from the intestine and circulated in the plasma. Caffeic acid has shown good pharmacological effects in clinical treatment and health care and is also used to prevent heart disease and reduce inflammation [2]. Caffeic acid has been vitally important as an ideal antioxidant to protect against the oxidative deterioration in many studies. Studies have shown that caffeic acid is used as an antioxidant to promote blood production and hemostasis. The results show that it had some toxic effects on the reproductive development of mice [3]. Animal studies have shown that caffeic acid prevents dietary hyperlipidemia and obesity by regulating the expression of liver lipid-producing genes [4]. Adipokines participate in the pathogenesis of atherosclerosis by promoting the inflammatory mechanism of insulin resistance. Lipid resistance causes endothelial dysfunction and plays an important role in the maintenance of atherosclerosis [5]. In addition, caffeic acid can effectively reduce uric acid levels, and studies have shown that caffeic acid and xanthine can competitively bind oxidase to inhibit its activity. By regulating the mRNA transcription of the renal uric acid transporter, it plays the role of lowering uric acid [6]. The antioxidant properties of caffeic acid make it important for energy conservation. Oil oxidation is an important factor affecting the quality of biofuels. Due to the presence of some unsaturated fatty-acid esters, oxidative rancidity occurs. As an antioxidant, coffee acid can improve the utilization of biodiesel [7]. Therefore, it is of great significance to monitor the concentration change of caffeic acid. There are many methods for the application of caffeic-acid detection, such as fluorescence technology [1,8], the QCM method [9], high-performance liquid chromatography [10,11], electrochemistry, and so on. Electrochemical biosensors have the advantages of fast detection speed, simple operation, high sensitivity, and easy miniaturization. However, complicated sample pretreatment and expensive test prices are not conducive to application, especially basic research, and the sensitivity and specificity of the caffeic-acid sensor is not great [12,13,14,15,16]. At present, the direction of detection research mostly exists in the analysis of plant-extract samples and agricultural products or blood and urine samples. Reported studies have shown that the samples required for different studies and the concentration of pharmacological analysis are mostly around 0–500 μM [2,17,18]. Therefore, it is still an urgent necessity to improve the application and performance to meet the needs of cheap and highly sensitive rapid detection of caffeic acid in various disease mechanisms, metabolic toxicology, and drug development. The construction of a biosensor interface is an effective and important approach that is inseparable from the composite of functional materials.

In recent years, multi-walled carbon nanotubes (MWCNTs) have attracted wide attention due to their large surface area, high strength, strong chemical stability, and conductivity [19,20,21]. They are widely used in electrochemical sensors. MWCNTs have unique physical and chemical properties and are one of the most popular materials for biosensor-interface modification. This is mainly attributed to their contribution to electron transfer and their large surface area, which can be widely used in the immobilization of biomolecules [5,22,23]. As we know, MWCNTs are not excellent in terms of solubility and dispersibility in water. Therefore, some biomacromolecules optimize performance of MWCNTs as a dispersant.

Chitosan (CS) is a kind of good biocompatible and biodegradable polymer that presents large amounts of amino and hydroxyl groups, so it can modify the surface of carbon nanomaterials in the process of synthesis [24]. At the same time, the exposed amino group from chitosan can also bind with the oxygen-containing functional group of caffeic acid so as to improve its detection performance [25]. The polymer has excellent film-forming ability and can provide many organic functional groups for functional modification. Multi-walled carbon nanotubes are prone to stacking and agglomeration, which not only affects their electrical conductivity, but also makes it difficult to use their functional modification sites. The chitosan–MWCNT composite can effectively improve the dispersion properties of carbon nanomaterials, leading to an increase in surface area and an improvement in electrochemical properties [26,27,28].

Protoporphyrin (PPIX) has many carboxyl groups and can provide many binding sites, so we modified the protoporphyrin in the amino side chain of chitosan by amide cross-linking to improve the catalytic performance of modified materials. In addition, protoporphyrin can chelate metal ions to form metal-centered protein structures and act as redox catalysts in many biological processes, such as electron transfer or oxidation of various organic substrates [29]. Protoporphyrin of chelated metal has catechol-oxidase activity and catalyzes the reaction of catechol functional groups of caffeic acid to some extent [30]. In addition, the metal ions in protoporphyrin are also sensitive to different degrees of interaction. This improves the electrochemical activity and is beneficial to the detection of caffeic acid by the sensor interface of composite materials.

In view of the importance of caffeic-acid detection and the need for higher detection performance, this study mainly used electrochemical-sensing technology to detect caffeic acid. The dispersion of multi-walled carbon nanotubes was improved by modifying chitosan. The exposed amino group from chitosan can bind with the oxygen-containing functional group of caffeic acid, and it can modify the surface of carbon nanomaterials with large amounts of amino and hydroxyl groups so as to improve its detection performance. The electrochemical properties of the sensing interface were enhanced by chelating metal-ion protoporphyrin, which has catechol oxidase activity and catalyzes the reaction of catechol functional groups of caffeic acid to some extent and forms an O-quinone derivative. Zn–PPIX–CS–MWCNT composite materials were successfully synthesized and modified on the surface of a glassy carbon electrode. The sensor interface was innovatively constructed and applied to the detection of caffeic acid for the first time.

## 2. Materials and Methods

### 2.1. Chemicals and Reagents

Caffeic acid, protoporphyrin IX (PPIX), chitosan, N, N-dicyclohexyl carbodiimide (DCC), bicarbonate, D-glucose, uric acid, L-ascorbic acid, acetaminophen, ferric chloride, cobaltous chloride, nickel chloride hexahydrate, cupric chloride, zinc chloride, and manganese (II) chloride tetrahydrate were purchased from Sigma-Aldrich (Saint Louis, MO, USA). N, N-dimethylformamide (DMF), and MWCNTs (30–50 nm diameter, 2 μm average length and 95% purity) were purchased from Aladdin. Acetic acid, KCl, ethanol, K_3_Fe[(CN)_6_], Na_2_HPO_4_, and citric acid were purchased from Tianjin Damao Chemical Reagent Factory (Tianjin, China). All the chemicals and reagents were of analytical-reagent grade and used as received without further purification. Deionized water was used throughout the experiments unless otherwise indicated.

### 2.2. Apparatus and Measurements

Electrochemical experiments were carried out on a 283 Potentiostat-Galvanostat electrochemical workstation (EG&G PARC with M270 software, Microsoft Windows XP) with a conventional three-electrode system, including the modified Pt electrode as the working electrode, a platinum wire (1 mm diameter) as the counter electrode, and an Ag/AgCl electrode (saturated with KCl) as the reference electrode. Transmission electron microscopy (TEM) image analysis was performed on a Tecnai G2 F20 instrument (Philips, Amsterdam, The Netherland). The EDX analysis was carried out on an energy-dispersive X-ray spectroscopy (EDX) analyzer that was equipped on the Tecnai G2 F20 instrument (Philips, Amsterdam, The Netherland). Fourier-transform infrared spectroscopy (FTIR) was conducted on a Bruker TENSOR37 (BRUKER, Karlsruhe, Germany) spectrometer with the KBr pressed-pellet transmission mode. Raman spectra were obtained with a Bruker RFS 100/S (BRUKER, Karlsruhe, Germany) spectrometer.

### 2.3. Preparation of CS-Functionalized MWCNTs

The CS-functionalized MWCNTs were prepared in conformity to the previously reported approach with a small modification [31]. A total of 10 mg MWCNTs was dispersed in 10 mL chitosan solution (0.01 g chitosan dissolved in 10 mL 1% acetic acid solution, pH = 2), and the blend was treated under an ultrasonic field for 30 min and magnetic stirring for 1 h. The product was then centrifuged at 10,000 rpm for 30 min and washed three times. Finally, the precipitate of CS–MWCNTs was dried suspended in acetic-acid solution at 1 mg/mL.

### 2.4. Synthesis of the PPIX–CS–MWCNTs

Two mg PPIX and 5 mg DCC were dissolved in 20 mL of DMF solution, and then 5 mL CS–MWCNTs were added to the mixed solution. The mixture was heated at 50 °C for 12 h with magnetic stirring, and the product was centrifuged at 20,000 rpm for 30 min three times. Finally, the precipitated PPIX–CS–MWCNTs were synthesized.

### 2.5. Synthesis of Me–PPIX–CS–MWCNTs

After dissolving 1 mL 0.1 M FeCl_3_, CoCl_2_, NiCl_2_, CuCl_2_, ZnCl_2_, and MnCl_2_ solution and adding an equal volume of PPIX–CS–MWCNT solution, the mixture was subjected to ultrasonic reaction at 65 °C for 3 h and centrifuged at 20,000 rpm for 30 min three times. Finally, the final product was Me–PPIX–CS–MWCNTs (Me = Fe (III), Co (II), Ni (II), Cu (II), Zn (II), Mn (II)).

### 2.6. Preparation of Me–PPIX–CS–MWCNTs/GCE

**Scheme 1:**
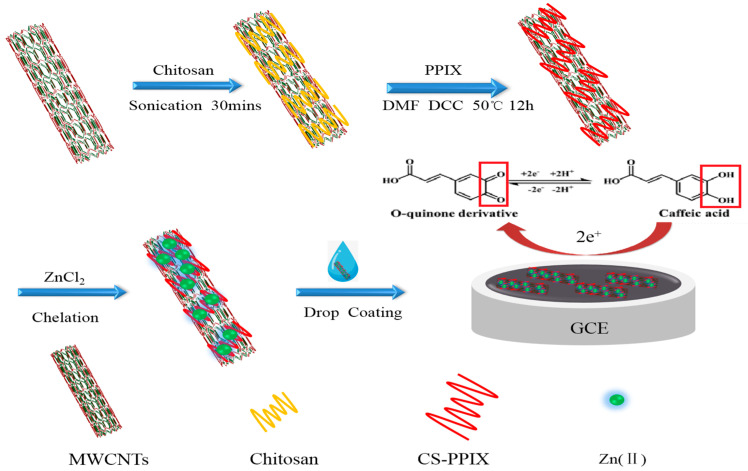
Illustration of the preparation of Zn–PPIX–CS–MWCNTs/GCE.

Firstly, putting GCE in the solution of ultrasonic processing containing sulfuric acid removes impurities, and then, powder polishing and processing was carried out from big to small in turn on different particle diameters (0.3 μm, 0.1 μm, 50 nm). Then, the GCE was alternately placed in anhydrous ethanol and deionized water, and ultrasonic cleaning was carried out for 20 min in order to eliminate the physical adsorption material. The above steps were repeated three times. Lastly, the cleaned GCE electrode was modified by 10 µL of Me–PPIX–CS–MWCNTs (Me = Fe (III), Co (II), Ni (II), Cu (II), Zn (II), Mn (II)) and dried naturally. The modified GCE electrode was used directly to detect for caffeic-acid sensors. The fabrication process is shown in Scheme 1.

## 3. Results and Discussion

### 3.1. Screening of Transition Metal Ions of Me–PPIX–CS–MWCNTs

**Figure 1:**
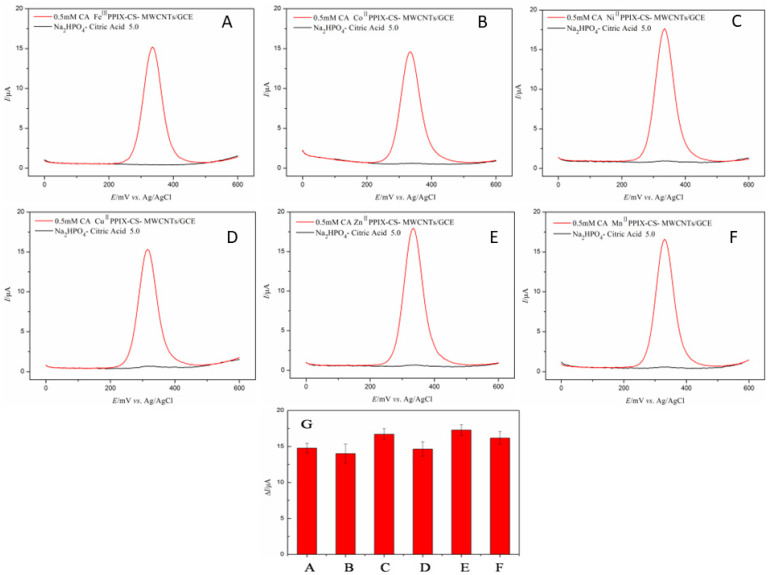
DPV of unmodified GCEs and GCEs modified with different Me-PPIX-CS-MWCNTs nanocomposites in 0.05 M Na_2_HPO_4_–citric acid solution (pH 5.0) containing 0.5 mM caffeic acid. (**G**) Histogram of oxidation-peak currents of (**A**–**F**). (Error bars = ±standard deviation, *n* = 5).

In order to study the electrochemical response of the GC electrode modified with different Me–PPIX–CS–MWCNTs ((A)FeIII, (B)CoII, (C)NiII, (D)CuII, (E)ZnII, (F)MnII) nanocomposites to detect caffeic acid, the DPV behaviors of the bare GC electrode and modified GC electrode were investigated in Na_2_HPO_4_–citric acid solution (pH 5.0) containing 0.5 mM caffeic acid (Figure 1A–F). Different modified GCE nanocomposites all presented an oxidation-peak current at 300–400 mV. Taking the curve of the bare GC electrode result as a baseline, the oxidation-peak current difference of the GC electrode modified with different Me–PPIX–CS–MWCNT nanocomposites was calculated (Figure 1G). Caffeic acid was determined to be highest for the oxidation-peak current of GC-electrode modified with Zn–PPIX–CS–MWCNTs. Therefore, we selected Zn–PPIX–CS–MWCNTs as the best GC electrode-modified nanocomposite to detect caffeic acid.

### 3.2. Characterization of Zn–PPIX–CS–MWCNTs

**Figure 2:**
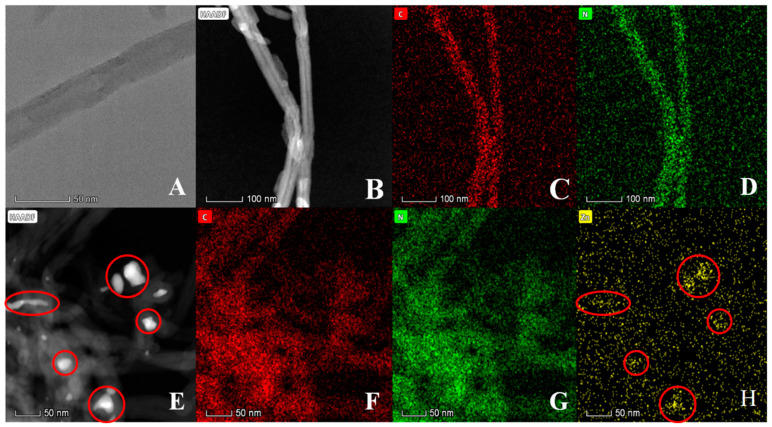
(**A**) TEM image of MWCNTs; (**B**,**E**) HAADF images of Zn–PPIX–CS–MWCNTs; (**C**,**D**,**F**–**H**) map-scan image of Zn–PPIX–CS–MWCNTs.

The morphology, characteristics, size, and crystallinity of the nanocomposites were determined using TEM, XRD, and FTIR. Figure 2A shows irregular hollow tubular structures of the MWCNTs, and the diameter was about 30 nm. Figure 2B refers to the HAADF image of the Zn–PPIX–CS–MWCNT nanocomposites, which shows that CS, PPIX, and Zn were modified on the MWCNTs. Figure 2C,D are the mapping scanning of the nanocomposite. The result shows that the distribution of elements C and N was concentrated on the MWCNTs. Element N was from materials of CS and PPIX. It shows that PPIX–CS–MWCNTs was synthesized. Figure 2E–H is the HAADF and map scan of the area of concentration material in the field of view. Figure 2F,G show the distribution of elements C and N. Figure 2H shows that the element Zn modified PPIX–CS–MWCNTs. Furthermore, the red circle of Figure 2H shows the concentration distribution of element Zn corresponding to the location of the red circle in Figure 2E. This demonstrates that the red circle of Figure 2E is the result of PPIX–CS–MWCNTs chelating element Zn.

**Figure 3:**
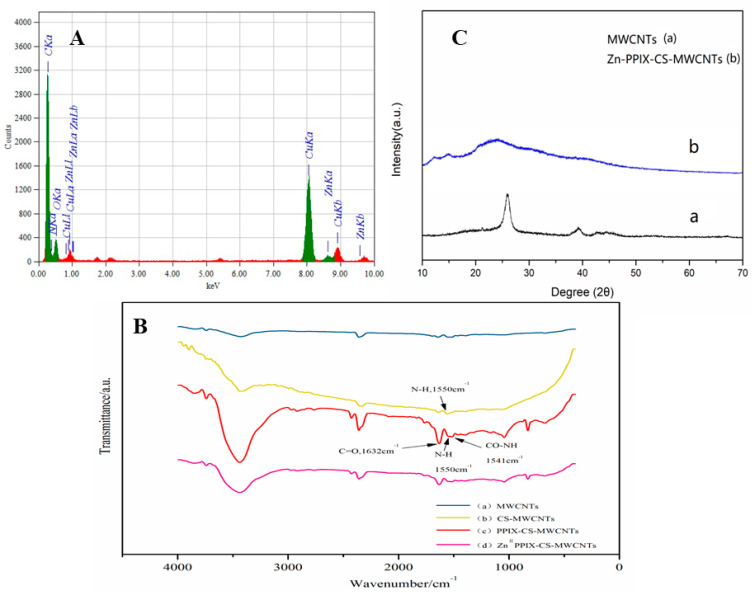
(**A**) EDX spectrum of Zn–PPIX–CS–MWCNTs. (**B**) FTIR spectra of MWCNTs (a), CS–MWCNTs (b), PPIX–CS–MWCNTs (c), and Zn–PPIX–CS–MWCNTs (d). (**C**) XRD patterns of MWCNTs (a) and Zn–PPIX–CS–MWCNTs (b).

The EDX analysis indicated that the nanocomposite Zn–PPIX–CS–MWCNTs consisted of C, N, O, and Zn elements. The Cu peak was from supporting the substrate membrane. These results indicate that the nanocomposite Zn–PPIX–CS–MWCNTs was synthesized. Furthermore, FTIR spectroscopy was applied to characterize the structure of the Zn–PPIX–CS–MWCNT nanocomposite. As shown in Figure 3B, curve b contrasted with curve a at an increased intensity of N-H (1550 cm^−1^), which was from the introduction of CS. Curve c increased the intensity of CO-NH (1541 cm^−1^) because PPIX formed amide bonds with CS through an amidation reaction. As shown in curve d, when Zn was introduced to PPIX–CS–MWCNT, all groups decreased the intensity of the stretching peaks. The XRD findings are shown in Figure 3C. Comparing curve a with curve b, the results show that the strongest and sharpest diffraction peaks of MWCNTs were around 2θ = 25.86°. At the same time, the MWCNTs sample also had small diffraction at 2θ = 39.02°. After mixing chitosan and Zn–PPIX with MWCNTs, many peaks appeared based on the diffraction peaks of the MWCNTs. A decreasing peak meant that the overall performance of the composite nanomaterials improved. In conclusion, the nanocomposite Zn–PPIX–CS–MWCNT was synthesized successfully.

### 3.3. Electrochemical Performance and Electrocatalytic Activity of Zn–PPIX–CS–MWCNTs

**Figure 4:**
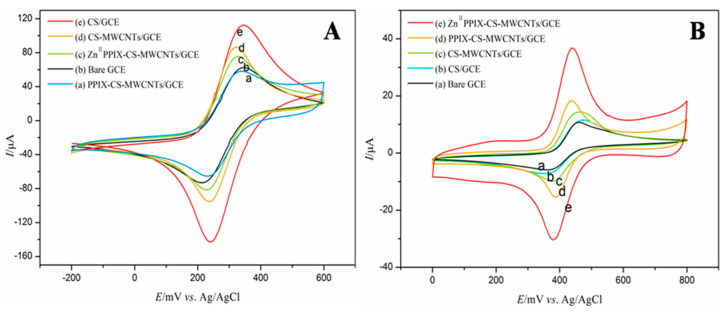
(**A**) Cyclic voltammograms of bare GCE (a), PPIX–CS–MWCNTs (b), Zn–PPIX–CS–MWCNTs (c), CS–MWCNTs (d), and CS (e)–modified GC electrode recorded in 0.1 M KCl solution containing 10 mM [Fe(CN)_6_]^3−^, scanning rate: 50 mVs^−1^. (**B**) Cyclic voltammograms of bare GCE (a), CS (b), CS–MWCNTs (c), PPIX–CS–MWCNTs (d), and Zn–PPIX–CS–MWCNT (e)–modified GC electrode in 0.05 M Na_2_HPO_4_–citric-acid solution (pH 4.0) containing 0.5 mM caffeic acid.

Different materials that modified GC-electrodes had a certain charge transfer and effective active surface area, and it was scientifically significant to apply cyclic voltammetry of the ferricyanide system for investigation. To compare GC electrodes modified with several materials, bare GC electrodes and nanocomposite modified GC electrodes were investigated in a 0.1 M KCl aqueous solution containing 10 mM [Fe(CN)_6_]^3^^−^ ions, and the result is shown as Figure 4. All modified GC electrodes represented a couple of redox peaks, which shows that modified GC electrodes have fast electron-transfer efficiency. The redox peak of the GC electrode modified with CS (curve a) was the highest, because CS is a good chelating metal ligand. Even though CS as a polymer does not have electrical conductivity, when chelating Fe ions it showed good electrical conductivity. The amino group in the C2 position of the CS molecule formed a cage structure with the adjacent hydroxyl group. Therefore, CS can compete with CN^−^ for Fe^3+^, leading to enrichment of Fe^3+^ on the electrode surface. It makes it easier to transfer electrons between the Fe^3+^ and the electrodes. When the carboxyl group in PPIX and the amino group in CS were bound by the amide bond (curve b), CS lost its ability to chelate Zn ions, and the result is like a bare GC electrode. When the Zn ion was introduced to the structure, the electrical conductivity of Zn–PPIX–CS–MWCNTs (curve c) was significantly increased. The results indicate that the Zn–PPIX–CS–MWCNT nanocomposite could be formed by a PPIX closed-loop conjugate structure chelating Zn ions, and its electrical conductivity was significantly higher than that of other materials that modified GC electrodes. The effective active area of different modified electrodes can be calculated by the Randles–Sevcik equation [32]:

Ip = 2.69 × 105A n^3/2^D^1/2^C_0_*v*^1/2^

Ip represents the redox-peak current in ampere; A is the effective active area of GC electrode modified with different materials; n relates to the number of GC electrons transferred during the redox reaction, *n* = 1; D is the molecular-diffusion coefficient (6.70 ± 0.02 × 10^6^ cm^2^s^−1^) in the reaction solution; C_0_ is the concentration of probe molecules (10 mM) in the reaction solution; and *v* relates to the scanning rate of the process (50 mVs^−1^). According to the above values, the effective active areas of electrodes modified with different materials can be calculated. The effective active area of the Zn–PPIX–CS–MWCNT-modified GC electrode was 1.20 higher than the bare GC electrode.

In order to investigate the electrocatalytic activity of Zn–PPIX–CS–MWCNTs we applied cyclic voltammetry (CV) in 0.05 M Na_2_HPO_4_–citric-acid solution (pH 4.0) containing 0.5 mM caffeic acid at a scan rate of 50 mVs^−1^. As shown in Figure 4B, the bare GC electrode (curve a) and the CS-modified GC electrode (curve b) had similar electrochemical response to caffeic acid because two phenolic hydroxyl groups of caffeic acid experienced the oxidation reaction at the same time. When CS (curve c) and PPIX (curve d) were introduced to the structure of MWCNTs, the electrocatalytic response was improved successively, which indicates that CS and PPIX have a certain catalytic effect on caffeic acid. When Zn ions were introduced to the conjugated polymers (curve e), the modified electrode obviously improved the oxidation-current response to caffeic acid. This indicates that Zn ions play a key role in catalytic caffeic acid. the redox peak was at +378 mV and +440 mV, which indicates that the reversible redox process of caffeic acid catalyzed by Zn ions occurred on the surface of the electrode.

### 3.4. Scanning-Rate Optimization and pH-Gradient Optimization of the Zn–PPIX–CS–MWCNTs/GCE Electrode

**Figure 5:**
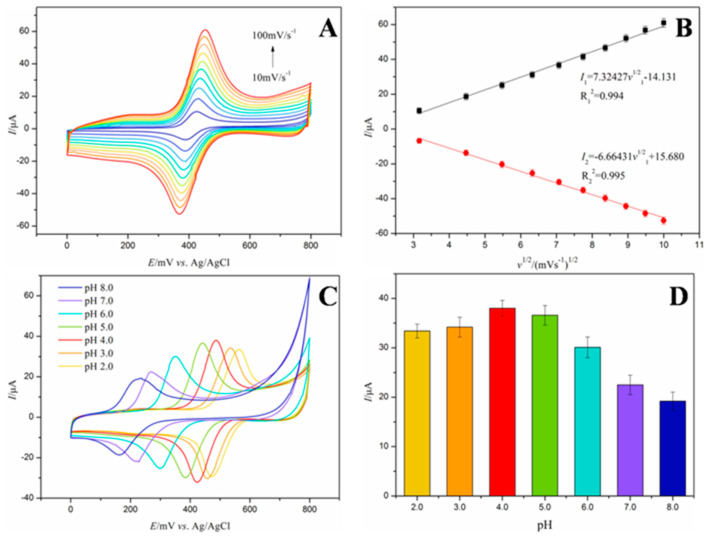
(**A**) Cyclic voltammograms of the Zn–PPIX–CS–MWCNTs/GCE toward 0.5 mM caffeic acid in 0.05 M Na_2_HPO_4_–citric-acid solution (pH 4.0) with different scanning rates. (**B**) Linear relationship of the oxidation–peak currents (Ip) and the reduction current (Ip) versus the square root of the scan rate (*v*^1/2^). Error bars = ±standard deviation, *n* = 5. (**C**) CV responses to 0.5 mM caffeic acid in different pH conditions with Zn–PPIX–CS–MWCNTs/GCE. (**D**) Linear relationship of the oxidation potential versus the pH. Error bars = ±standard deviation, *n* = 5.

Different scanning rates affected the electrocatalytic activity of Zn–PPIX–CS–MWCNTs-modified GC electrodes by CV, which was observed in 0.05 M Na_2_HPO_4_–citric-acid solution (pH 4.0) containing 0.5 mM caffeic acid. The result is as shown in Figure 5A, and the scanning rate ranged from 10 mVs^−1^ to 100 mVs^−1^. As the scanning rate increased, the oxidation- and reduction-peak currents gradually increased. The oxidation- and reduction-peak currents at different scanning rates were linearly fitted to the square root of the scanning rate, and two linear-fitting equations were obtained, as shown in Figure 5B. The linear-fitting equation of the oxidation- and reduction-peak currents were determined to be I_1_ = 7.32427*v*^1/2^ − 14.131 (R^2^ = 0.994) and I_2_ = −6.66431*v*^1/2^ + 15.680 (R^2^ = 0.995), which suggests that the electrochemical redox of the Zn–PPIX–CS–MWCNT-modified GC electrode to caffeic acid is a diffusion-controlled process. According to the Randles–Sevcik equation, the diffusion coefficient of caffeic acid is 6.78 × 10^−5^ cm^2^s^−1^. In order to ensure the stability of the catalytic experiment, we selected 50 mVs^−1^ as the scanning rate of all test experiments.

In addition to the optimization of the scanning rate, the pH value of the detection environment had a great influence on the redox activity of caffeic acid. In order to investigate the optimal pH value of the Zn–PPIX–CS–MWCNT-modified GC electrode, the result was observed via CV in Na_2_HPO_4_–citric-acid solution (pH 2.0–8.0) containing 0.05 mM caffeic acid. As shown in Figure 5C, as the pH value decreased, the oxidation peak potential of caffeic acid gradually moved to negative potential and the maximum oxidation-peak current appeared at pH 4.0. The oxidation potential corresponding to the maximum oxidation current of caffeic acid was fitted linearly to the pH value and the linear fitting equation was expressed as E = −57.10 pH + 635.714 (R^2^ = 0.991), as shown in Figure 5D. The absolute value of slope 57.10 mV was close to the Nernstian theoretical value (0.059 V), which indicates that the number of protons and electrons is equal in the electrocatalytic oxidation of caffeic acid. Therefore, pH 4.0 was selected as the optimal reaction pH value in all experiments.

### 3.5. Electrochemical Detection of Caffeic Acid by Zn–PPIX–CS–MWCNT-Modified GC Electrode

**Figure 6:**
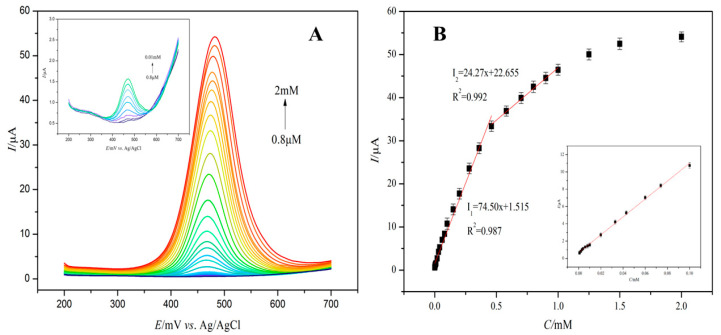
(**A**) DPVs of the Zn-PPIX-CS-MWCNT-modified GC electrode with different concentrations of caffeic acid from 0.8 μM to 2 mM; (**B**) calibration curve of the electrocatalytic oxidation-peak current as a function of caffeic-acid concentration.

Differential pulse voltammetry (DPV) is an electrochemical method with high resolution to measure the high sensitivity of substances. Based on the above studies, DPV was used to study the relationship between current response and different concentrations of caffeic acid in 0.05 M Na_2_HPO_4_–citric-acid solution at pH 4.0 in order to investigate the electrocatalytic properties of the Zn–PPIX–CS–MWCNT-modified GC electrode. The result is presented in Figure 6. With the increase in concentration from 0.8 μM to 2.0 mM of caffeic acid, the oxidation-peak-current value increased gradually in the range of 200 mV–700 mV. The oxidation-peak current was fitted linearly to the concentration of a series of caffeic acids, and the result was obtained with two linear-regression equations within the range of 0.8 μM–1.6 mM. Within the range of 0.8 μM–1 mM, the linear-fitting equation was expressed as I_1_ = 74.50x + 1.515 with a correlation coefficient of 0.987, and in the range of 1 mM–1.6 mM, the linear-fitting equation was presented as I_2_ = 24.27x + 22.655 with a correlation coefficient of 0.992. According to the slope of the two linear-regression equations, the sensitivity of the caffeic-acid biosensor was 886.90 µAmM^−1^ cm^−1^ (74.50 µAmM^−1^) in this study, and the limit of detection (LOD) was calculated as 0.022 μM based on S/N = 3. In order to compare the performance of different modified electrodes, we listed other modified electrodes and Zn–PPIX–CS–MWCNTs/GCE with linear range and detection limit in Table 1. The results show that Zn–PPIX–CS–MWCNTs/GCE showed the lowest sensitivity compared to others, and the linear range was also wider than most modified electrodes. This was due to the optimized performance of the electrode after dispersing the MWCNTs with CS and chelating the Zn ion, which increased the electron-transfer efficiency and catalytic capacity between the caffeic acid and the electrode.

### 3.6. Study on Repeatability, Stability, Selectivity, and Applicability of Caffeic-Acid Sensors

**Figure 7:**
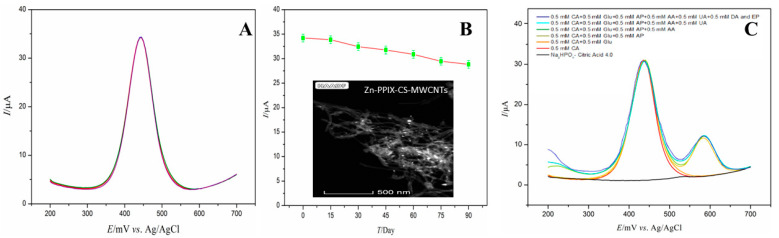
(**A**) Five GC electrodes modified with Zn–PPIX–CS–MWCNTs were prepared by the same procedure and determined in 0.05 M Na_2_HPO_4_–citric-acid solution (pH 4.0) containing 0.5 mM caffeic acid. (**B**) The response of oxidation-peak current of the same GC electrode modified with Zn–PPIX–CS–MWCNTs was determined to be 0.5 mM caffeic acid at an interval of 15 days. Error bars = ±standard deviation, *n* = 5. (**C**) Interference test of the fabricated biosensor in Na_2_HPO_4_–citric-acid solution (pH 4.0) containing 0.5 mM caffeic acid and the same concentration of interfering substances.

Reproducibility and stability are indicators of good performance of biosensors. We investigated the oxidation-peak-current response of five GC electrodes modified with Zn–PPIX–CS–MWCNTs prepared by the same procedure in 0.05 M Na_2_HPO_4_–citric-acid solution (pH 4.0) containing 0.5 mM caffeic acid. The result is shown in Figure 7A and it can be seen that the oxidation-peak current of the five electrodes were almost identical and the RSD was approximately 4.49%. This shows that the modified electrode had good reproducibility in this study. The response of the oxidation-peak current of the same GC electrode modified with Zn–PPIX–CS–MWCNTs was determined to be 0.5 mM caffeic acid at an interval of 15 days. After 90 days, the oxidation-peak current reached 84.3% of the initial current. This result shows that the biosensor constructed in this study has good stability (Figure 7B). The insert in Figure 7B shows the morphological analysis of the composite material, which exhibited a good structure compared with that before the sensing. The agglomeration of the composite appeared slightly, which may be related to the reduced stability caused by the electrochemical reaction.

What is more, selectivity ability is also an important indicator of caffeic acid biosensor performance. In order to evaluate the selectivity of the fabricated biosensor, the electrocatalytic activity of the caffeic acid and electron-transfer interference of some substances was studied, and 0.5 mM caffeic acid was detected by DPV in the presence of the same concentration of interfering substances. The result is shown in Figure 7C. Glu had no effect on the oxidation peak of caffeic acid, but the oxidation peak of 0.5 mM AP and AA appeared at 585 mV and 220 mV, respectively, and the oxidation peak of UA was about 220 mV. What is more, the oxidation peak of DA and EP was around 200 mV. The addition of all interfering substances had no effect on the oxidation peak of caffeic acid, indicating that the fabricated electrode prepared in this study has good anti-interference performance.

In order to verify the applicability and reliability of the caffeic-acid sensor, the Zn–PPIX–CS–MWCNTs/GCE was applied to the determination of caffeic acid in Na_2_HPO_4_–citric-acid solution of a real drug sample, as shown in Table 2. We can see that the recovery ranged from 98.0% to 101.0% and the results were roughly the same as the data found by HPLC. All consequences show that Zn–PPIX–CS–MWCNTs/GCE displayed excellent application.

## 4. Conclusions

In this study, Zn–PPIX–CS–MWCNTs were successfully synthesized by modifying protoporphyrin on the amino side chain of chitosan and chelating Zn ions. An excellent caffeic-acid sensor was prepared by using the outstanding performance of metalloporphyrin. Finally, the nanocomposite-modified electrode exhibited good catalytic activity, high sensitivity, a wider detection range, lower LOD, better stability, repeatability, and selectivity, with easy operation. Based on the good experimental results of this experiment, applying other small molecular monomers (pyrrole, aniline, etc.) that have the conjugate structure or small water-soluble monomers (acrylamide, etc.) to combine metalloporphyrin as sensitive modification elements to construct biosensors with excellent performance for the detection of biomarkers can be proposed. This provides a new idea for electrochemical-interface construction and biomarker detection, and also provides a foundation for the study of the best performance of different metal chelation on various biomarkers in future research.

## Figures and Tables

**Table 1:** Comparison of electrochemistry biosensors modified by different nanomaterials for the detection of caffeic acid.

Electrode Materials	Linear Range (μM)	Detection Limit (μM)	Reference
CDs@MIPs	0.5–200	0.11	[33]
Au@a-Fe2O3@RGO	19–1869	0.098	[14]
Nafion/ER-GO	0.1–10	0.091	[12]
AuNP−CHIT	0.05–2000	0.025	[34]
laccase-MWCNT-chitosan	0.735–500	0.275	[35]
Zn-PPIX-CS-MWCNTs	0.8–1600	0.022	This work

**Table 2:** Measurement results of caffeic acid in Na_2_HPO_4_–citric-acid solution.

Sample	CA Added (μM)	HPLC Found (µM)	CA Found (µM)	Recovery (%)
1	0	3.15	3.17	100.6
2	10	13.15	13.30	101.0
3	20	23.10	22.80	98.7
4	30	34.80	34.10	98.0
5	40	42.50	42.90	100.9
